# Extracts and Gleanings

**Published:** 1876-09

**Authors:** 


### Extracts and Gleanings.

---

*Cicuta in Scarlet Fever.*—Dr. Fiske (*Western Lancet*, July, '76) reports several cases of scarlet fever treated with cicuta. Among the number we select the following:

In July last I was called to see the child of Mrs. C., on Stockton street, age three years. I found its face flushed, the skin hot and dry, throat sore, with considerable tumefaction on the right side, tonsils swollen, elongated, and covered with a whitish exudation, amid which could be seen ragged edges of mucous membrane; while the projecting papapillæ of the tongue and its dry, coated condition, together with a fine scarlet eruption which was very profuse over the face and chest, all told, in unmistakable terms, the nature of the disease. The child was very restless, had a dry hacking cough, considerable nausea, and a diarrhœa with quite offensive discharges. There had been several cases of scarlet fever reported in the neighborhood previously, with one or two deaths. My patient had not, however, been brought in contact with these. The diagnosis was, of course, scarlet fever. My first prescription was to quiet the nervousness and irritability, which were quite distressing and persistent. I prescribed the chalk mixture and opium, for the double purpose of giving relief to the diarrhœa and to the excitement. The next day there was some improvement in the above mentioned symptoms, but evident signs of uræmic poisoning had now developed themslves, the discharge of urine for the past forty-eight hours having been quite scanty, and the passage of the catheter failing to reveal the presence of any in the bladder. Becoming alarmed at the critical condition, I suggested consultation, but was told that they had confidence in me and would prefer that I should continue as before. I then gave small doses of calomel and digitalis, ⅛ of grain of former to 1-16 of the ext. of the latter, every two hours, with directions to continue the same until purging took place, or until I should again see the patient.

The next day there was no improvement; uræmic symptoms more pronounced. The cellular tissue of the abdomen and hips was quite distended with serum. The patient was restless, with more or less diarrhœa and some flatulence; moaning, unconscious, feet and hands cold. The eruption disappearing, I prescribed fl. ext. cicutæ gtts. xxx, syr. simpl. oz. iij. *M.* Sig. Give one teaspoonful every three hours. Hot application to the extremities and an infusion of wild cherry bark—according to U. S. P.—one tablespoonful given every three hours. The next day the flow of urine had sensibly increased, and the anasarca diminished. The action of the heart, which I forgot to mention, had been very irregular by reason of what appeared to be a distended condition of the pericardium, was now much improved. The brain was clearer, and the whole condition much more satisfactory. From this time onward the patient continued to improve till convalescence was fully established. In this case the beneficial action of the cicuta was so marked that it was noticed by the friends; and the mother especially, who was a very intelligent woman, always mentioned that bottle and its charming effects with tears of gratitude.

---

*The Electrolytic Treatment of Tumors.* By JULIUS ALTHAUS. (London.) Berl. Kl. Wochenschr., Allg. Med. Central. Zt., 35, 1876.

In consideration of the great difference in opinions about the therapeutic usefulness of electrolysis, the author gives a brief account of his latest observations in this field.

1. *Nævus.* It is most frequently found in the face and on the scalp, and electrolysis is the most appropriate means for its removal. It is superior to excision, because there is no hemorrhage; to injections of sesquichloride of iron, because it does not endanger the life; to the cauterization by nitric acid, because it can be localized better; to the subcutaneous ligation, because after the operation the child has no pain and the surgeon has no further trouble; over the galvanocaustic, because it does not leave any scar. The ordinary round, small and thin nævus usually yields to one single application, while the larger "portroine stains" require a number of sittings.

The current acts through needles, which set in rows, and connected with the poles of ten to fifteen Daniell's elements are thrust into the tissue of the nævus. As soon as the chain is closed the destruction of the texture begins with the blood vessels and the integument of the nævus appearing to wither. The destruction being more thorough around the positive pole, this must always be applied to the worst portion of the nævus. Usually not a single drop of blood is lost; if, however, by any sudden motion of the patient, a needle should come out, and a drop of blood should appear, this can be coagulated at once and any further bleeding can be prevented by directing the positive pole to the puncture. When all the morbid tissue seems to be destroyed, the current is interrupted and the surface of the nævus is covered by a piece of gold beater's skin. No further dressing is necessary, because the surface remains dry. The eschar is thrown off after about ten or fourteen days, and the surface gradually assumes the appearance of the neighboring integument.

2. *Goitre.* The writer considers electrolysis as especially applicable to the cystic goitre, because it occasions neither pain nor danger. The best *modus operandi* is to introduce two or three needles into the cyst and to connect the same with the negative pole, while the positive electrode is applied to the integument by means of a wet sponge. Through the electrolytic action caustic soda is created out of the solution of chloride of sodium contained in the cyst. This caustic soda cauterizes the secretory lining of the cyst and stops any further secretion. Two to six applications usually are sufficient. In the treatment of parenchymatus goitre the author combines the electrolysis with injections of tincture of iodine into the tissue of the goitre.

These parenchymations injections break up the texture of the tumor, and thus the combined method shortens the time of treatment quite materially, especially if the goitre is old and rigid.

3. *Sebaceous Tumors.* They can easily be excised; but many patients dreading an operation, and the excision being occasionally followed by dangerous, even fatal, erysipelas, Althaus advises the use of electrolysis, which will remove these tumors without danger and without leaving a scar. Both electrodes are put into the tumor to have a quick result.

4. *Carcinoma.* The writer advocates the early and thorough extirpation of every primary cancer. But in cases of secondary carcinomata the electrolysis has often done a good service. It cannot, of course, eradicate the cancerous diathesis, nor prevent the fatal termination, but it is very efficient in allaying those lancinating pains, in procuring sleep and comfort to the patient.

The histories of cases are given in support of the author's views expressed in the paper.—*Chicago Medical Journal and Examiner.*

*Electricity, Its Therapeutic Applications.* — (*The Practitioner,* 1876.) The electric force in all forms is a disturbing element, and is therefore unsuited to the conditions in which *rest* is the ndication.

It may be assumed that this agent is inapplicable in all cases of recent or acute inflammation. After the subsidence of the acute symptoms, however, this agent may be beneficial. The same is true of friction and vibration. The appreciation of this principle will save some disappointments from the misapplication of the remedy.

Headache from acute inflammation or acute congestion, will, according to this principle, be aggravated by electricity. A headache from inanition or anæmia will be benefitted, and sometimes instantly removed by electricity. A sympathetic headache will be removed by this agent when the sympathetic cause is removed.

A dyspeptic headache will generally prove intractable, because the gastric derangement will defy removal by this agency.

On the other hand, a headache dependent upon defective in nervation in the sympathetic or ganglionic system, will disappear by the electric stimulation of the sympathetic ganglia. In this case it is not necessary to include the brain within the scope of the current.

In the chest "a stitch in the side," or any form of neuralgia is generally benefitted, while a true pleurisy is made worse. In doubtful cases, therefore, the behavior of the agent may be made a means of diagnosis.

In the abdomen, constipation from sluggish muscular action and deficient intestinal secretion is benefitted by this agent, while the same symptoms resulting from inflammatory action are only made more intense. The intestinal muscles paralyzed by inflammatory congestion are still more congested by the electric stimulus.

In the loins, the condition of lumbago is favorable for the beneficial action of this agent, while a deep-seated inflammation is unfavorable.

In the pelvis, especially in the female, the organs are benefitted or injured, according as we comply with this principle, or neglect or misunderstand it.

A congestion dependent upon local muscular passiveness in the walls of the vessels ought to be benefitted, while a true acute or recent inflammation will be aggravated. The practical appreciation of this agent should, upon this basis, be an aid to diagnosis.

Sciatica follows the rule of lumbago, while an inflammation of a nerve presents a condition unfavorable for the application of electricity.

An exception may be made in the apparent effects of a powerful galvanic current, which is capable of producing a benumbing effect, even in an inflammatory condition. In this case the apparent benefit is temporary, the pain soon becoming as bad as before.

*Odorless Iodoform.*—Ether dissolves iodoform and robs it of its unpleasant odor. Applying this solution of it, leaves it in a uniform SMELLLESS coating on the surface receiving it after the evaporation of the ether.—*Chicago Med. Jour. & Exam.*

*Diphtheria Treatment.*—Z. C. McElroy, M. D. (*W. Va. Med. Student*, May, 1876,) says:

"For the bulk of cases, where the temperature does not remain high, or does not reach higher than 103° or 104° F., I seldom make more than one prescription, and am not often called to supplement it by other measures, except in severe cases, by some opiate to relieve headache, or something to open the bowels. In the latter case I do so by half a grain calomel granules, one every six hours, the desired evacuation almost always occurring within twenty-four hours. The prescription almost always used for an adult is about as follows:

| | | |
|---|---|---|
| R | Chlorate Potass | dr. j. |
| | Dilute Alcohol, } *aa.* | f dr. vj. |
| | Pure Water, } | |
| | Tinc. Capsicum | f dr. iij. |
| | Liq. Ferri Perchlor. | f dr. j. |

Sig.—A teaspoonful in a wine-glassful of water, to be used as a gargle each time.

After gargling, a teaspoonful in a tablespoonful of water, more or less, to be slowly swallowed, and repeated every three to six hours.

This simple plan of treatment may be varied by increasing or decreasing the quantity of the Perchloride of Iron. For very young children sometimes only five to ten drops, to the two ounce mixture, seldom exceeding the drachm called for in the prescription, for adults, preferring to increase or decrease the frequency of its administration. The Liq. Ferri Perchlor. is not the muriated tincture of iron, though the preparations are both perchlorides. I do not get the same results from the tincture as I get from the solution, and, therefore, do not now ever prescribe the tincture, though I have thought druggists sometimes use it under the impression that they are identical. I have often to write on prescriptions, "not tincture of iron." I have no doubt there are cases occurring in this and other localitles requiring other agents, as Quinia, milkpunch, etc., but within the limits of my practice I do not observe them. Diet, bread and milk, to be taken whether there is appetite or not.

I think it a little remarkable that I get very small children to use this gargle; not unfrequently they ask for it when the time draws near to take it. They say it makes their throats feel better. It saves them from having brushes or swabs put in their throats, a proceeding to which they never fail to object."

*Spider Web or Tela Araneæ in Chronic Intermittents.* L. M. JONES, M. D., (*Cin. Lancet and Observer*, May, 1876), says:

Being at his wit's end, in treating a rebellious case of intermittent in a girl ten years old, with *quinine*, *camphor chinoidine pills*, *arsenic*, *eucalyptus*, *iodine*, phosphoric acid, etc., etc., with unsatisfactory results, Dr. Jones determined, upon Dr. Robert Jackson's recommendation in the U. S. Dispensatory, to use spider web. He then says:

"Accordingly I went to the drug store and had some prepared in the following manner: Went with the druggist to the cellar (as it is the species of spider that inhabits cellars or dark places, that possesses medicinal properties) and with a stick I gathered cobwebs until I had a wad or bunch the size of a large hulled walnut.

"This we put in a bottle with four ounces of good whisky, which was allowed to macerate for forty-eight hours, when it was filtered, and the liquid poured into a bottle. I carried the medicine to my patient, and left the following directions: Begin four hours before the expected chill, and give a teaspoonful every hour, until she had taken four doses, then a teaspoonful before each meal and at bedtime, until all was taken. The medicine was given as directed. The anticipated chill came, but was very light compared with the others. This, however, was the last chill that she had, which has been over four months since.

"Her general appearance has improved, color of skin is clear, the spleen has returned to nearly its natural size, bowels regular, appetite good; in fact, the child has so far improved that her friends call her well.

"The patient and relatives did not know what she was taking, and are still ignorant of the subject, so that the mind of the patient had nothing to do in the performance of the cure.

"I gave her the second bottle of the medicine, to be certain that the cure was effectual.

"I have since used the remedy in other cases, with the same success, and would ask that the profession give the remedy a trial in the various intermittents. I will offer no comments on the case that I have cited, or give any theory as to the action of the remedy, but shall leave the reader to his own views. I give a history of this case because I considered it an interesting one in several particulars; also was particular in giving the treatment, that a comparison might be made in the remedies used, and the results obtained; and that the reader might see for himself that the treatment adopted by the writer was a varied one. I will add, in conclusion, that either of the prescriptions given will break up an ordinary case of intermittent fever, chronic or not."

*The Symptoms and Prognosis of Cerebral Anæmia.*—In the West Riding Lunatic Asylum Reports, Dr. J. Milner Fothergill speaks of this disease:

The symptoms of cerebral anæmia are numerous. Dilatation of the pupil is "one of the commonest accompaniments of cerebral anæmia." Pallor of the face is very frequent, pallor of the eye a still more valuable indication, especially where the ophthalmoscope reveals an anæmic condition of the disc and retina. This is distinguishable from atrophy of the optic nerve by "the uniform grayish-white appearance of the disc, and the fact that one disc is in exactly the same condition as the other." The expression of the face is peculiar. "There is a mingled look of sadness and general impairment of expression." There may be corrugation of the brows and depression of the angles of the mouth. The general state of the circulation is most important. There is an adynamic condition of the heart, and the arteries are diminished in volume, the pulse being feeble and compressible. The surface is reduced in temperature, the hands and feet being cold, and often blue, from venous congestion. The skin is often dry, withered and wrinkled. There is drowsiness by day and sleeplessness at night. The position of the head, it being erect during the day and recumbent at night, may have something to do with this. Headache is a very frequent symptom, being of a dull, persistent, unvarying kind, and usually vertical; frontal headache being rather associated with passing conditions of exhaustion from sustained intellectual labor. Vomiting, in the graver cases, may be produced by the patient suddenly sitting up. This may be due to sudden anæmia of the roots of the vagus causing the stomach to contract, as there is no nausea nor action of the abdominal muscles. Palpitation of the heart may similarly be caused by the vagus ceasing to act. Sighing respiration is frequent. Constipation, so common in cerebral affections, may be either a cause or a consequence of cerebral anæmia. The general muscular condition is one of relaxation and impaired power, the patient being "listless, unenergetic and easily exhausted." In the earlier stages there may, however, be a condition of restlessness and irritability. There may be various lesions of sensation, local or general anæsthesia, dullness of the special senses, and even hallucinations. The psychical symptoms are loss of mental power, melancholic depression, and, in the more pronounced cases, dementhia In the early stages there may be great irritability, and in some instances this is the chief symptom. There is frequently a feeling of being "cabined, cribbed, confined," either by some supernatural or natural power; or there may be some delusion with respect to attempts at poisoning, etc. In many cases the anæmic condition of the brain produces that craving for stimulants known as dipsomania.

The prognosis is favorable in simple cases, which do not depend upon grave physical disease. Those cases which have their starting-point in some mental shock are more favorable than those in which physical disease is the primary factor; while those which are complicated with dipsomania are among the most unfavorable.

*Gastric Vertigo.*—The following excellent article is by Dr. C. A. Brice in the West Virginia *Medical Student:*

I would call particular attention to this trouble, which is almost always associated with and dependent upon an accumulation of gas in the stomach. This is a most distressing trouble and until the patient fully realizes his exact condition, and that his ultimate recovery is certain, he will be perfectly miserable, thinking he is going to be paralyzed, or that he has serious disease of the brain. I must thank my friend and former professor, Dr. L. S. Jaynes, for relieving my mind, and making a very correct diagnosis of my own case, when I was a great sufferer from this real disease. The patient will hardly know how to describe his feelings. It is not literally a vertigo, but a general foolish feeling; want of power to think closely and correctly; and this condition is frequently associated with perverted vision. These symptoms come on suddenly, thus alarm the patient and prevent him from feeling safe in walking, or trusting himself alone; and they leave suddenly. He lives in constant fear of an attack, which is always returning, making him lose confidence in himself, and depressing his spirits until after a while life itself is a burden. As a medical man, I have experienced all of this in my own case, and have since recognized and treated it in my patients. Many a poor dyspeptic has run the gauntlet of every imaginable drug, thus weakening the powers of the stomach and aggravating his complaint, for the want of a proper diagnosis. I have been thus particular respecting gastric vertigo because the books are deficent upon the subject. I have never been able to find anything that fully described my own case, or any general case of the kind.

Concerning the treatment of this complaint I will say that the long and faithful use of strichnia, charcoal, pepsin, and calcined magnesia did me more good than anything else. Of course my diet was cautiously regulated, and I have found that prudence in eating and drinking constitutes the most efficient treatment for this class of dispeptics. The peculiar mental condition in such cases is enough to make us investigate the diseases fully. Many a patient suffers for a long while before mentioning it to his medical attendant. I have had patients tell me, confidentially, that they feared losing their minds, and would go on and give the exact symptoms of gastric vertigo, which would always yield to treatment; but I will say that in no case have I seen anything do good speedily; nothing but strict attention to diet, and the prescribed remedies kept up, sometimes for months, can relieve the patient.

There is this peculiar feature of the trouble: it is worse in the morning and evening. A patient will be unfit for any physical or mental effort until 10 or 11 o'clock in the day, and for the next several hours he experiences relief. Toward sunset, however, his trouble returns.

*Amyl nitrite in Nervous and Mental Diseases.* PROF. PICK. (*Irenfreund*, 2, 1876.)—The physiological effect of an inhalation of amyl nitrite is a dilation of the arteries, especially of the head; a diminution of the arterial pressure, and an acceleration of the action of the heart. The psycological effect of the remedy is manifested by a hilarious mood of the patient. The effect lasts but a few minutes, and leaves no unpleasant sensations, unless an impure preparation was used. The increased frequency of the contractions of the heart is due to the diminution or total abolition of the influence of the pneumo-gastric nerve on the functions of the heart. The dilatation of the arteries is caused by the direct influence of the amyl nitrite in the blood on the muscular tunic of the vessels. The amyl nitrite has successfully been employed in all those nervous and mental diseases which are caused by muscular contraction of the arteries or by an excessive arterial pressure.

*Hemicrania*, attended by hard pulsating temporal arteries, and by extreme paleness of the face, was quickly relieved by the inhalation of amyl nitrite.

*Epilepsy*, caused by an anæmia of the brain, as the result of arterial spasms, yielded readily to the inhalation of amyl nitrite. If there was an "aura" preceding the convulsions, the attack could almost always be prevented by an inhalation. Brunton first recommended the amyl nitre for *angina pectoris*, and its favorable influence on this affection has since been confirmed by many writers.

As to its therapeutic value in melancholy the statements are very conflicting. Meynert claims to have obtained favorable results from its use, while others do not think their patients have been benefitted by it.

*Diagnosis of Fissures of the Anus in Children at the Breast.*—Marboux, in *L' Union Medic.*, No. 61.)—Three affections—hæmorrhoids, polypus of the rectum, and fissure of the anus, are accompanied by pain and hæmorrhage, in connection with defecation. Exploration of the anus is indispensable in order to arrive at a diagnosis. This should be performed with great delicacy, so as not to increase the rent (when it exists), and especial care should be taken in completely separating the folds of the mucous membrane, for fissure is always found between two folds, and, when small and recent, is liable to be overlooked in a superficial examination.

Polypus of the rectum is ordinarily seen when the anal opening is examined at the moment of defecation, in the form of a small, red, mamelonnated enlargement, somewhat resembling a raspberry. External hæmorrhoids are readily seen, but then the folds of membrane should always be separated for inspection, as fissure may also be present. Internal hæmorrhoids may also be seen through the anal orifice in the efforts of defecation; and sometimes are with difficulty, and only after several examinations, dissociated from polypus. If exploration of the anus gives negative results (neither external hæmorrhoids nor fissure), and there are general symptoms pointing to rectal lesion, the diagnosis is between internal hæmorrhoids and polypus beyond recognition by ordinary exploratory procedures. Hence other means of recognition must be adopted.

An important sign of fissure is the unusual resistance encountered by the finger in overcoming the sphincter. When the finger has been previously well oiled, and is introduced as gently as possible, and yet the infant cries with pain, the existence of fissure may almost be affirmed, and the characteristic lesion is almost always encountered.

*Specific against Hydrophobia.* GRZYVALA. (*British Md. Jour.* April, 1876.—The writer claims for XANTHIUM SPINOSUM antirabic properties. Its efficacy has been tested in one hundred victims bitten by rabid animals, of whom he lost none. Some astonishing instances of the marvelous power of this drug are given, two of which are appended. Twelve persons of one family had been bitten by a mad wolf. Six of this number were admitted into the hospital of Olschanka, Government of

Podolia, district of Balta, and were treated with this drug, and all recovered. All of the others, treated with the actual cautery and the daily use of *genista tinctoria* died with hydrophobia in from twelve to sixty days. Thirty oxen had been bitten by a mad wolf; five of them died hydrophobic. The remaining twenty-five were treated with XANTHIUM SPINOSUM and recovered. Of the dried leaves, powdered, the dose for an adult is nine grains, thrice daily. For children under that age, half that dose. For the animals above alluded to, the dose was three ounces daily, given in bran. Too warm a welcome to this new aspirant for the honors of specificity against hydrophobia cannot be extended. The trustworthiness of Dr. Grzyvala is vouched for by Prof. Guber, of Paris.

(XANTHIUM SPINOSUM, or CLOT WEED, is a native of the United States as well as Europe, growing in the fields and roadsides from Massachusetts to Pennsyvania and Georgia. It is a plant growing about one foot high, very conspicuously armed with straw-colored spines, and possessed of distinctly sudorific properties.—*Ed. Jour. and Exam., Chicago.*

*Treatment of Placenta Prævia.*—In a paper on this subject, published in the *American Practitioner* for June, Dr. Parvin, of Indianapolis, advises, in conformity with the teachings of Greenhalgh and Thomas, the induction of premature labor, and expresses a belief that the mortality of both mothers and children, in cases of placenta prævia, will undergo a marked diminution when this is adopted by the profession as a rule of practice. He considers Barnes' dilators to be the safest and best means for the induction of the premature labor, and they moreover bring it on more rapidly than any other means. The vaginal tampon is difficult of application ; is uncomfortable to the mother ; does not remove the possibility of a dangerous internal loss of blood, and possibly may lead to a separation of the placenta and death of the child. Ergot is objectionable, except when the os is well dilated or dilatable and the labor can be speedily terminated, for the tetanic contractions it excites are apt to asphyxiate the child. Puncture of the membranes is obviously dangerous for the child, and as far as the mother is concerned is not free from danger, as it may possibly change an open into a concealed hemorrhage. Podalic version increases the risk to the child's life, and probably may be limited almost if not quite altogether to cases of shoulder presentation. Complete separation of the placenta, as advised by Sir James Simpson, is a method which ignores the child's interests, and has never received any general professional support. Finally, the partial detachment urged by Dr. Barnes does not seem to be a rational mode of treatment, for it simply increases the bleeding surface.—*N. Y. Medical Record.*

*New Treatment for Tricophyton.*—At the meeting of the *Societe Medicale des Hopitaux*, on May 26th, M. Lailler read, in the name of M. Lespian, a paper on the treatment of parasitic and non-parasitic affections of the skin. In 1875, while stationed in the Pyrenees, M. Lespian saw a number of dogs that were suffering from a parasitic affection that bore considerable resemblance to tinea: scaly cutaneous eruptions, the hairs glued together and breaking off close to the roots, and slaty color of the skin. His own two dogs were affected, and on microscopical examination he found almost always spores. Within a short period, thirty-four persons, twenty-eight children and six adults, were affected with the same disease, the parts affected being those most frequently in contact with the hands. The treatment employed by M. Lespian consisted in painting with the following mixture: R. Tannin, grs. xv; tincture iodine, dr. iss; glycerine, dr. v. M. When the disease was not very extensive, two applications daily for four days generally produced a cure. Pre-existing crusts were softened by the application for twenty-four hours of wads of cotton wadding or charpie soaked in olive oil or glycerine. M. Lespian has sometimes seen fever, pytalism, and laryngismus occur during the treatment, which probably resulted from iodism.—*La France Medicale*, May 31st, 1876.—*New York Medical Record.*

*The Indications for Quinine in Surgical Practice.*—(Verneuil—*Gaz. Med.*, *Med. Times & Gaz.*, 1876.)—M. Verneuil states that quinine may be beneficially employed in treating ataxic, neuropathic and septicæmic complications. 1. It is not necessary for the ataxic phenomena to be of a true intermittent type, for quinine exercises its regulative action when no palustral element exists. 2. It is especially valuable in combating neuropathic phenomena, and nowhere can its influence be better appreciated than in affections of the eye. M. Verneuil is in the habit of prescribing it after operations on this organ, and he has frequently obtained most remarkable results. 3. In septicæmic accidents the salutary action of quinine is beyond all doubt, and given in large doses it still constitutes the best agent we have to oppose to purulent infection. 4. As an antiseptic it appears to act in two modes—modifying and diminishing the formation of pus, and operating as a direct antiputrefactive—so that it is advantageously employed as a topical antiseptic. 5. Its surgical employment is especially indicated for women and children, who are more disposed than men and adults to ataxic and neuropathic accidents.

*For Soap Liniment*, the use of castor oil soap has been advocated in some journals, which would, no doubt, answer the purpose admirably. The following modified formula of the "Pharmacopœia," with my experience has worked well:

| | |
|---|---|
| Take of Castile soap | 4 troy ounces, |
| water | 14 fluid ounces, |
| camphor | 2 troy ounces, |
| ol. rosemary | ½ fluid ounce. |
| strong alcohol | 1½ pint. |

Grate the soap on a common grater, and, having mixed the water with one-half pint of alcohol, pour this on the soap; with occasional agitation it will dissolve in a short time. Dissolve the camphor and oil of rosemary in remainder of alcohol. Add this to the solution of soap, let stand some time and filter. The result is a preparation which will not precipitate. It is not as strongly alcoholic as the officinal, but it contains as much alcohol as is compatible [? Ed.] with it as a preparation.—*Carl S. N. Halberg, PH. G., in American Journal of Pharmacy.*

*The Plethysmograph.*—This is the name of an instrument invented by Dr. Mosso, of Turin, to measure the action of the blood vessels in the human body. The apparatus is described as a sort of muff, made of vulcanized India-rubber, somewhat similar to the apparatus employed by Junod for dry-cupping. This is filled with water, at the temperature of the body, and so arranged as completely to envelop the member to be experimented on. The water in the muff is made to communicate with a cupel, by means of two glass tubes, which are equally filled with water, suspended over another liquid of less density. As soon as the slightest motion occurs in the muscles or blood-vessels of the limb (the forearm, for instance), the water rises or falls according to the degree of motion imparted to it. In other words, if the blood-vessels of the forearm be dilated, the water in the tube rises; if contracted, the water falls. A needle fixed to one of the tubes traces an interrupted line, which faithfnlly represents the succession of movements imparted to it through the liquid.—*Med. & Surg. Rep.*

*Topical Treatment of Chronic Dysentery.*—The reporter narrates three cases of topical treatment of dysentery, followed by cures. They were of several months' standing each. The first case, that of a girl fourteen years old, was of six months' duration. She was in a very low condition, with a pulse 130 and scarcely perceptible, skin covered with a clammy sweat. Her body had emitted a cadaveric odor for several days; death seemed inevitable. After etherization, a bivalve speculum was introduced into the rectum and the "mucous membrane was found highly inflamed and studded over with small yellowish ulcers, which, on slight pressure, emitted a colored fluid." Silver nitrate was freely applied to every part of the bowel, as high up as could be reached with the aid of a retractor. This application was followed by an ability to control the bowel. The appetite was improved, strength increased, and recovery of the vital powers was very speedy. Daily injections of carbolic acid solution (one part to eight of water) were used. In two weeks the patient made a complete recovery. The other two cases were similarly treated, and recovery followed.—*M. Dills, M.D., of Carlysle, Ky., in N. Y. Med. Jour.*, April, 1876.

*Intestinal Atony.*—For asthenic constipation, the writer recommends ergot and phosphorus; the latter apparently enhances the action of the former. Ergot, it is well known, is a vaso-motor stimulant; vascular contraction follows its ingestion, and this partial anæmination produces muscular contractions; hence the increased peristalsis. This formula is recommended:

R. Fl. Ext. Secal. Cornut...................dr. vij

Acid Phosphorici Diluti.................. ..dr. j

M. S. Teaspoonful three times a day. — *Canada Medical Record.*

*Acute Bright's Disease—Sialagogue Effect of Jaborandi.*—The case presented certain peculiarities which made it worthy of note. When admitted, only 7½ to 11½ ounces of urine were passed in twenty-four hours, and it contained albumen, epithelial and granular casts. The patient was dry-cupped over the kidneys; received a cathartic, composed of croton oil, podophyllin, and elaterium, and dram doses of the fluid extract of jaborandi. The latter was administered for the purpose of producing diaphoresis, and soon after the second dose was given the patient broke out in exceedingly profuse perspiration, and, in addition, the remedy acted as a sialagogue to such a degree that oz. xxii of saliva were collected in four hours. At the time of our visit the patient was passing oz. lx of urine in twenty-four hours, acid, had a sp. gr. of 1023, and contained no albumen. After the operation of the cathartic, the infusion of digitalis with acetate of potash was administered.—*Bellevue Hospital Medical Record, N. Y.*

*Sulphurous Acid in Enteric Fever.*—Thirty cases were treated with sulphurous acid, in doses ranging from three to fifteen drops, in lemonade, every four hours. Only one patient of this number died; this one patient "was a fragile girl, whose life was gradually wasting away with consumption, but she recovered, then relapsed and died." The writer thinks the acid acts as a specific upon the fever poison, arresting at once its further development, and thus exterminates the fever. Amelioration ensues at once, and in a very few days the patients, under the influence of this agent, are convalescent. Within twenty-four hours the tongue becomes moist and commences to clean; the diarrhœa is speedily arrested, the tympanites subsides, the pulse slows and grows stronger, the digestive faculty speedily asserts itself, and the patient is soon out of danger.—*J. Wesley Botkin, M.D., Morrisonville, Ill., in Philadelphia Med. & Surg. Reporter*, May, 1876.

*Convulsions Arrested by the Sinistro-Lateral Posture*—Mr. Bader first pointed out to the profession the advantages of putting the patient on the left side when threatened with danger under chloroform inhalations. Knowing the good effects of this procedure, Dr. Brown applied it in case of a man with Bright's disease, in urecmic convulsions, and the latter ceased instantly. Again he resorted to the same expedient in a man seized with unilateral (right) convulsions, whose consciousness and speech were intact: he had been convulsed ten minutes when the doctor arrived, and immediately turned him upon his right side, and the convulsive action ceased in ten or fifteen seconds. What does this mean? Similar successes are greatly desired to confirm a startling fact—if this postural treatment resulting curatively *be* a fact.

*Hyposulphite of Soda in Diphtheria.*—The remedy not only tends to diminish the temperature, but to destroy the cryptogam of the false membrane. The hyposulphite of soda is given in doses of from 6 to 20 grammes in from 100 to 300 grammes of distilled water, to which 30 grammes of syrup of orange peel are added. At the same time a gargle is administered, containing 40 grammes of the hyposulphite of soda in 400 of distilled water. The diet should consist of eggs, soup and wine. During convalescence the prolonged use of lactate of iron is recommended. Dr. Tamborlini reports numerous successful cases thus treated.

*Gelseminum.*—Pain and weakness in both arms of two years' standing, arising from piano playing, were cured by taking 8 drops of tincture of gelseminum thrice daily for three weeks. In cases of neuralgia of the tregeminal, supraorbital, brachial and sciatic nerves, this drug, in 5 to 20 drops of the tincture, thrice daily, has been successfully used. In some cases it fails utterly, but in a larger proportion of cases of the neuralgias it can be reliably used.

*Acute Articular Rheumatism—Ice Treatment.*—Two cases were seen, which illustrated not only the benefit derived from this plan of treatment, but represented two classes of patients; namely, those in which the joint symptoms are accompanied by only slight constitutional disturbance; and those in which the constitutional disturbance becomes the leading feature.

The ice treatment was regarded as the one which afforded special benefit in those cases in which the joint symptoms are prominent, and the success of the treatment depends very much upon the thoroughness with which it is carried out. The object is to relieve pain; and this is done very effectually by the application of ice, if it is properly used. It should be powdered (snow answers equally as well), and placed in ice-bags made of rubber or oil silk, and these should surround the joint affected. If the ice is not powdered, the joint is apt to be only partially covered, hence the application is not thorough. Properly applied, it seems to be almost a specific against the pain of this affection. The ice-bags should remain around the joint for 36 or 48 hours, and then they may be removed, but should be reapplied as soon as the pain returns.

The patient should be kept in the house just as long as when under any other treatment; and the usual duration of the disease was from 14 to 21 days. While ice was used, no other treatment was given; and by its use the patients before us were very effectually relieved of pain, which is decidedly worth accomplishing in a rheumatic patient.

Another case was seen, in which was illustrated the opposite condition already mentioned; namely, one in which the constitutional symptoms were the prominent feature. There was high temperature, some delirium, and evidently some constitutional disturbance. In this case, the patient was placed in a bath, having a temperature of 80° or 90° F., kept in for ten minutes, and the bath was repeated in two hours with great benefit.

There was another case, the like of which every practitioner has seen, one in which the disease had lasted for a long time; the patient had become anæmic, and, although the joints were involved, they were not acutely so. The patient was being helped out of his difficulty, as many of this class of cases can be—not all, however—by the use of the following pill, taken three times a day:

R. Strychnine....................................gr. $\frac{1}{30}$.

Quinine, sulph..........................grs. iij.

M.

Ft. pil.

—*Bellevue Hospital—Medical Record.*

*Liquor Patassæ in Diphtheria.*—In a letter to the June number of the Boston *Journal of Chemistry*, Dr. Edward H. Sholl, of Gainesville, Alabama, says:—

Some five or more years since, my attention was called to an article on this subject in my weekly companion, the MEDICAL AND SURGICAL REPORTER, of Philadelphia, by a physician of Philadelphia, whose name I do not now recall, directing attention to the use of the liquor potassæ in this disease.

Not satisfied with any treatment pursued in my practice prior to that time, the resolution was made to test this. An opportunity was soon afforded in a case of an adult male, and of extreme severity. To be certain, four physicians were called to examine and diagnose the case. All agreed as to its specific nature. For more than twenty-four hours the disease had been treated with iron, chlorate of potash, ammonia, etc., but the symptoms of debility, with local invasion of the throat, were rapidly increasing. All previous medication was suspended, and he was put upon the use of the liquor potassæ alone, in twenty-drop doses every three hours. In thirty-six hours every trace of the membranous deposit was gone, and the fever subsiding. The patient went on to speedy convalescence, and was soon able to leave my office, where I had kept him in order to conduct the experiment accurately. Since that time the remedy has been used, with like result, in every case of diphtheria coming under my care, and is given in doses suitable to age, every three hours. Usually, in the early stage, I alternate it with a four-ounce saturated solution of chlorate of potash, to which is added one fluid drachm of hydrochloric acid and two of tincture of iron, of which a small teaspoonful, properly diluted, may be given to a child six years old every three hours, allowing thus an hour and a half between the different medicines. When the membrane disappears, the iron mixture is discontinued, and an emulsion of cod-liver oil and syrup of lacto-phosphate of lime used till strength is restored. The liquor potassæ is continued as long as the membrane is present, and until the fever entirely gives way.

*Chronic Ulcers of Leg.* Dr. Turney (*Ohio Med. and Surg. Jour.*) remarks: Over two years ago I was treating one of those intractable ulcers on the leg of a woman of more than sixty years. The sore was of many years standing, situated over the internal malleolus, oval in shape, about two inches in its long diameter, with hard, thickened, everted edges, and a base utterly destitute of healthy granulations. The leg nearly to the knee was covered with a fiery red, sweating, eczema. The veins were turgid and varicose. I had tried the changes on ordinary and extraordinary medication, with no apparent improvement. During this time I had occasion to use Esmorch's anæmic bandage for the removal of a tumor from a leg. In using this I was struck with two things: First, the complete bloodlessness and dryness of the tissues; and second, the marked hyperæmia which soon followed its removal. Indeed, the frequent occurrence of troublesome parenchymatous hemorrhage after the use of this bandage is to me the only objection to its surgical employment. These observations led me to the use of the bandage in the case of chronic ulcer I was then treating. I saw that the veins would be not only emptied of their load of impoverished and vitiated blood, but would be refilled with a fresh charge of oxygenated, nutritive blood. I applied the elastic bandage as tightly around the limb as high as the knee, as if I had intended to do a bloodless amputation. I left it on as long as the patient would bear it, say abour ten minutes. This was repeated once a day. No other treatment, local or constitutional, was pursued. The patient, a woman of active habits, was allowed to go about her ordinary avocations. In a few days improvement was marked, and the ulcer went steadily to rapid and permanent healing in less than three weeks. So firm is the cicatrix that it has resisted a subsequent attack of acute eczema.

Since then I have used the same method of treatment in eight intractable ulcers of long standing—one in a woman of seventy years, of twenty-five years' continuance. The daily application of the elastic bandage in the manner described; no other treatment, local or constitutional, was given—no confinement to bed or elevation of the member involved. They have all made rapid and permanent cures.

*Prophylactic Treatment of Puerpural Fever.*—Professor Bischoff uses carbolic acid for this purpose. He employs it in a regular antiseptic treatment just as surgeons do in operative cases. His aim being to remove all noxious matter before the genitals are wounded, and to prevent any infectious matter from entering these parts after they had received some lesion. Prof. B. enforced the following plan of antiseptic treatment in his lying-in hospital in Basil: At the commencement of labor pains the woman receives a bath and a vaginal injection of a lukewarm aqueoms solution of carbolic acid (strength two per cent.) And these injections are repeated every two hours in all cases of protracted labor, of artificial premature labor, and of dead fœtus. Before making an exploration or operation, the hands and instruments must be washed in a three per cent. solution of carbolic acid and lubricated with a carbolized glycerine ointment, or a carbolized oil containing 10 per cent. of carbolic acid. Immediately after the delivery the external parts are examined and all wounded places are touched with the carbolized oil. Lacerated wounds of the mucous membrane of the vagina, and those frequent, though superficial, rents near the orifice of the urethra, are then united at once by the means of fine catgut sutures; larger lacerations of the perineum are closed by silver wire, and these sutures are left undisturbed until the woman can get up, *i. e.*, for two weeks. And it is a very rare thing, then, not to find these lesions healed by first intention. After all the wounds are closed, a pledget of charpie, well saturated with carbolized oil, is put in the introitus vaginæ, to be renewed as often as it drops out, *i. e.*, after urinating and vaginal injections. Besides this, a second and larger pledget of carbolized charpie is laid over the external fissures or cuts. And in all cases, from six to twelve hours after delivery a vaginal injection with a two per cent. solution of carbolic acid is made, and afterwards repeated two or three times daily; and if any coagula or pieces of the placenta are retained, the uterine cavity is washed out also by injections of carbolized water. These injections have never occasioned any untoward symptoms or had any poisonous effect.

*The Treatment of Pruritus Hiemalis.*—Dr. John Pirnat, M.D., (*Medical and Surgical Reporter*) says: As you say, in your 1st of January number, that you know of no efficient treatment for pruritis hiemalis, I take the liberty to forward to your journal the treatment for the disease which crowned with success four of my cases. The readers of the *Reporter* I refer to the *Half-yearly Compendium*, for July, 1874, where a good description of the above named disease can be found. The treatment I pursue is the following, viz.

R. Zinci sulph.............................dr. vj.
Ammonii muriat...........................oz. ivss.
Aquæ pluvialis.............................Oiv–vj.

Sig. Wash the body all over, morning and evening, till quite well.

R. Ext. colocynth comp. .......................dr. vj.
Mass. pilul. hydrargyris..................dr. ss.
Pulv. myrrhæ..................................gr. x.
Mucil. gum arab...............................q. s.

Fiat pil. No. 16.

Sig. One at bedtime every evening.

R. Kali iodat ......................................dr. ij.
Kali bromati.....................................dr. v.
Kali nitr...........................................dr. vj.
Extract buchu...................................f. oz. ij.
(or extract pareir brav......................oz. ij.)
Aquæ menth. pip.............................oz. ijss.
Aquæ lauro cerasi............................dr. vj.
Ext. conii fl.......................................dr. iij.

Sig. One desertspoonful, in half a tumbler of water, a half or one hour before meals.

I never failed yet with this combined treatment, viz: 1, the stimulating and astringent wash; 2, the alterative laxative pill; 3, the alterative, sedative and diuretic mixture, which will surely correct the perverted condition of the glandular structures, and so lead to a healthy nutrition of the sudoriferous glands, which are secondarily diseased.

[We are also informed that cosmoline, applied locally, has effected immediate relief, and apparently a cure.—*Ed. Rep.*]

*Treatment of Pruritus Vulvæ.*—Lotions containing alum may be used according to the following formula:

R. Aluminus..................................oz. i,
Decoct. hordei.................................O iv. M.

Apply three or four times a day.

M. Hardy employs the following formula:

R. Hydrarg. bichlor .....................gr. xv,
Aquæ destillat............................oz. iij, sc. v,
Alcoholis....................................q. s. M.

Sig. A tablespoooful in a tumbler of water. In applying, care should be taken not to rub the parts.

In that form of pruritus vulvæ which so often accompanies pregnancy, Dangan employs the following formula:

R. Zinci oxid.......................................dr. i,

Sodii borat.......................................dr. ij,

Cerat simpl.......................................dr. iv,

Ol. amygdal. dulc...............................q. s.,

Morphiæ muriat...................................gr. i. M.

M. Bazin prescribes in pruritus the following liniment:

R. Liq. calcis,
Glycerinæ, aa..........................dr. vijss,
Ol. amygdal. dulc.....................dr. i, dr. vi,
Ol. rosmarini vel gaultheriæ......gtt. v. M.

He also occasionally makes use of the following formula:

R. Glycerinæ.............................150 grammes,
Tinct. opii..............................4 grammes,
Ol. Rosmarini vel gaultheria...gtt. v.

—*L'Abeille Med.*—*New Remedies.*

*Atropia as an Antidote to Prussic Acid.*—The *Druggist's Circular*,

January 1st, contains a letter from Dr. T. B. Jackson, of Macon City, Mo., in which he says:

Two cases of poisoning by hydrocyanic acid, which lately occurred in this vicinity, caused me to make a number of experiments on dogs, with the object of studying the effects of the poison, and of trying various substances so as to find an antidote. In this, I think, I have been successful. Sulphate of atropia, in doses of one-fourth of a grain to one grain, injected under the skin, gave prompt relief in every case, even when large doses of the acid had been given. When the two poisons are administered at the same time, none of the effects of prussic acid are developed; but if as much as one grain of sulphate of atropia be injected, all the symptoms of atropia-poisoning are observed. In some instances the antidote was withheld until the animal would fall down, and the respiration would be as slow as six per minute, the dog being unconscious; then one-fourth of a grain of the antidote would relieve him instantly.—*Medical and Surgical Reporter.*

*Therapeutic Value of Picrotoxine.*—*Gazette Medicale de Paris*, No. 51, 1875.—According to recent researches, *picrotoxine* exerts a special action on the medulla spinalis and oblongata. M. Gubler has hence been led to employ it in a case of labio-glosso-pharyngeal disease, and obtained well-marked improvement after a few days. The patient, in fact, who, on admission, was only able to take liquid food, consumed the usual diet of the hospital; the salivation had disappeared, and speech had become more easy. Picrotoxine has been administered in doses of one milligramme (.01544 grain). At the point where the injections were made, small, hard, indolent tubercles, resembling syphilitic gummata, appeared, which slowly disappeared. This hyperplastic action of picrotoxine on the cellular tissue is, M. Gubler observes, interesting no notice. M. Dujerdin-Beaumetz has tried the effect of picrotoxine in epilepsy, beginning with smaller doses than the above, and attaining ultimately three milligrammes per diem. Here, too, prompt amelioration took place in the symptoms. The fits, which in the first instance occurred every day, only took place every second day, then became more and more rare, till they ultimately disappeared altogether. The patient quitted the hospital after two months, and had not again been seen, though he promised to return should the fits recur. This result, does not, of course, demonstrate that the epilepsy was cured, but simply that an attack had been staved off for two months; but it suggests that further trials should be made with it. No results have been obtained from the employment of picrotoxine in paralysis agitans.

*Syphilitic Headache and Neuralgia Cured by small and repeated Doses of Calomel.* PETER. (*Lancet*, July, 1875.)—The author gives the details of several cases treated in the manner recommended by Trousseau, viz: giving about one-sixtieth of a grain of calomel every hour. The interesting features of this method of medication, as described by Dr. Peter, are: 1. The rapidity of its action; 2. The fact of its success in cases where the really specific treatment of syphilis fails. It constitutes, in a manner, *the* medicine of nocturnal syphilitic pain, but cannot replace the other plan of treatment for other syphilitic manifestations. Its use is indicated whenever the pain is intense and induces asomnia. It diminishes pain and its consequences the very first night it is given, and generally extinguishes suffering by the second night. The treatment may be carried on for three days, and that period of time is almost always enough for its success. If, however, the desired result has not been obtained, it ought then to be suspended for one or two days, so as to prevent salivation, and it can then be resumed afterwards for two days successively, in which case Dr. Peter has never seen it fail.

The plan of treatment is thought to be very efficacious, because, 1st. The drug is mercury; and, 2d. The absorption of these very small doses is exceedingly rapid, and the repetition of the action takes place every hour. Whatever the action may be, adds Dr. Peter, it is to Trousseau that we are indebted for the idea of using calomel in this manner, and to him belongs all the credit.

*Action of Jaborandin, or Chlorhydrate of Pilocarpine.—Centralblatt, f. d. Chirurgie*, No. 52, 1875.—The alkaloid obtained from the summitates jaborandi (pilocarpus pinnatus), termed jaboran din, or, better, pilocarpin, has a more uniform action than the leaves themselves, and, according to Siredey, acts somewhat differently from them. The doses recommended by Dr. Dumas in his thesis are from half a grain to two grains, from which no injurious influence need be anticipated. The action on the salivary glands is the most marked, and commenced very soon after its administration, so that in the course of five hours a rapidly increasing, and then a slowly diminishing, salivation took place, especially from the submaxillary glands. The total excretion of saliva in this period amounted to 755 grammes (11,-657 grains, more than $1\frac{1}{2}$ lbs. avoir.). Copious sweating occurred one hour after the commencement of the salivation, which slowly diminished in the course of four hours. Billiary vomiting usually occurred, the urinary secretion was greatly diminished, the pulse was slowed, the temperature lowered, the pupil dilated. No therapeutic value has been yet assigned to it.

*Treatment of Epistaxis by the Internal Administration of Ergot.—British Medical Journal.*—Dr. George St. George, of Lisbon, observes that the treatment of epistaxis is often attended with great difficulty, especially in persons enfeebled by age. He has found ergot of use in cases where liquor ferri perchloridi, plugging and other remedies have been tried without avail. The following is one ef the cases he records: A weak anæmic woman, aged 55, had been suffering for three days from repeated and violent attacks of hæmorrhage from the nose, which had increased in both frequency and violence during the last twenty-four hours. The nostrils were first plugged with lint and cold water. Dr. St. George then tried plugging with lint dipped in liquor ferri perchloridi, but without avail, as the hæmorrhage continued. He then ordered her a mixture, each dose to contain fifteen minims, of liquid extract of ergot every quarter of an hour until the hæmorrhage ceased, and then to be continued every four hours for a day or two. In an hour and a half the bleeding had entirely ceased and never returned. He gives the details of two other cases successfully treated by the same means.

*Esmarch's Bandage for Chronic Ulcers.*—Dr. Turney, of Ohio, has employed this bandage in seven cases of ulcers of the leg, one a typical indolent ulcer, with indurated edges, over the internal malleolus of a woman over eighty-five years of age. In six cases the cure was rapid and permanent; in one a portion of the cicatrix gave way, but it was again progressing favorably when the patient disappeared. The bandage was applied firmly from the foot to the knee, once a day, and allowed to remain as long as it could be borne, about ten or fifteen minutes. No other treatment was employed. With each application oxygenated blood takes the place of a fluid unfit for nutrition; the strong pressure effectually overcomes the passive congestion and œdematous infiltration, and the distended vessels, completely relieved of their load of vitiated blood, have an oppurtunity to recover their lost tonicity.—*N. Y. Med. Red.*

*External Treatment of Urticaria.*—The following applications are used by Dr. Hardy at the Hopital Saint-Louis, in connection with appropriate internal remedies:

LOTION OF CHLOROFORM.

R. Chloroformi..................................dr. ijss,
Alcohol..........................................oz. ijss.

M.

LOTION OF CORROSIVE SUBLIMATE.

R. Hydrarg. bichlor........................gr. iss. ad iij,
Aq. destillat..............................oz. iij. sc. v,
Camyhoræ. ...............................q. s. M.

Sometimes starch-powder alone or in the following combination:

R. Amyli.............................................dr. x,

Zinci oxid......................................dr. ss,

Camphoræ.......................................dr. ss. M.

Apply morning and evening. Vichy water, alkalies, etc., may be serviceable at the same time to correct acidity of the stomach.—*New Remedies.*

*Homicidal Handwriting.*—Under this heading the London *Lancet* refers to the careless manner in which physicians are in the habit of writing their prescriptions, and gives a case in point: A gentleman received a prescription for a cough, one of the ingredients of which was benzole rect. By the merest good luck the recipe was properly compounded. Some time afterwards the patient presented the same prescription to another chemist, who read the ingredient referred to as benzoli *nit.*, remarking upon the largeness of the dose. The patient assuring the compounder that he had taken the dose named, without bad effects, it was given to him. The result was that he barely escaped with his life. The *Lancet* truly says: "The whole blame of the accident must be attributed to the prescriber. The practice of relying on a particular chemist to interpret bad writing is very objectionable, and cannot be too strongly condemned. Every prescription should be legible at a glance."—*N. Y. Med. Record.*

*Pomegranate Bark for Tape-worm.*—PROF. EDWIN FREEMAN, M. D. (*Ec. Med. Jour.*, May, 1876.)—I succeeded in obtaining a tape-worm entire, with its head, from W. S., of Avondale, which had resisted the use of a good many medicines during five years before I began to medicate its possessor for it. The worm obtained was thirty-four feet long when it came away. Mr. S. said that he passed, at some time past, forty-two feet in one piece, but the head remained, and he has every day passed several inches, for at least three years. While he had the worm he was a large eater, and seemed to require, besides, a quart or two of coffee every morning. It caused him, at times, to faint and dizzy, with occasional headache and weakness and sluggishness in the morning. In addition to this, there were occasionally nausea and fullness of the stomach. The best evidence of the presence of tape-worm is the passage, with the fæces, or portions of one.

*Treatment.*—I gave him, the first thing in the morning, two seidlitz powders, which thoroughly evacuated the bowels. I then gave him morphia sulph. gr. ⅛. In an hour he began to take the pomegranate, a decoction of the bark, four ounces every fifteen minutes, until it was all taken. The decoction was prepared by the chemist, J. U. Lloyd, from the best bark, according to the formula published by Prof. Locke, in a former number of the E. M. Journal, and mixed with fluid extract jalap, dr. j.

After the third dose the worm was felt to have lost his hold on the bowel, and to be low down. The fourth dose was not taken until an hour after the third, hoping that the worm would surrender and come away. It was stubborn, however, but at the last dose submitted unconditionally, like Davy Crockett's coon, coiled himself into a knot, and got down and out.

The dose is a fearful one to swallow, but for those who can take it, it is effectual in ridding them of a very annoying trouble.

*Preparations of Iodoform.*—Dr. J. H. Johnson, of Providence, R. I., sends (*Boston Jour. Chem.*) the following note: "Since the article on Iodoform appeared in the February number of the *Journal* so many letters have been received from your readers in the profession requesting the formulæ for the glycerole of iodoform and the iodoform suppositories, as I use them in practice, that I inclose them for publication: *Iodoform Suppositories*—Iodoform (in powder) 30 grains; oil of theobroma, 7 drams; triturate the iodoform with half a dram of the oil, add to the remainder the oil previously melted, and make six suppositories. *Glycerole of Iodoform*—Iodoform, 40 grs.; glycerine 1 ounce; triturate the iodoform with the glycerine. The suppositories have proved very efficient in inflammation and induration of the womb. The glycerole of idoform, employed for the same purpose, is applied upon pledgets of charpie saturated with the medicament."

*Recipe for Curing a Taste for Liquors.*—At the festival of one of our reformatory institutions a gentleman is reported to have said: "I overcame the appetite by a recipe given to me by old Dr. Hatfield, one of those good old physicians who do not have a percentage from a neighboring druggist. The prescription is simply an orange every morning half an hour before breakfast. 'Take that,' said the doctor, 'and you will want neither liqnor nor medicine.' I have done so regularly, and find that liquor has become repulsive. The taste of the orange is in the saliva of my tongue, and it would be as well to mix water and oil as rum with my taste." The recipe is simple, and has the recommendation that it can do no harm even if it does no good.—*Boston Journal of Chemistry.*

*Sage's Catarrh Remedy.*—According to Adrian Bowers, in the *American Journal of Phaımacy*, this popular nostrum may be reproduced very nearly by employing the following formula and manipulation :

Take of hydrastis canadensis, in powder......gr. v,
Indigo..............................................gr. ss,
Powdered camphor..................................gr. ij,
Carbolic acid.........................................gr. ij,
Sodium chloride.....................................gr. l.

Rub the camphor with the salt previously reduced to a moderately fine powder ; rub the indigo and carbolic acid together ; mix with the salt and camphor ; and, lastly, add the hydrastis, and mix intimately in a mortar, without much pressure.—*New Remedies.*

*Two New Sources of Lead Poisoning.*—In the *Gazette Med.*, of May 6, 1876, Dr. Gilbert called attention to two new sources of lead poisoning. In one case the editor of a newspaper had suffered for two years from severe gastric disturbances, the explanation of which was ultimately found in a lead line on the gums, and the supply of the poison was found in the red-lead coloring of the stamps which the patient moistened with his tongue every day in large numbers. In the second case the patient consumed so many of the little French *cahons*, such as are used by smokers, as to become poisoned by the lead in the metallic film with which they are "silvered." On chemical analysis each box was found to contain about a third of a grain of lead.—*New York Medical Record.*

*Treatment of Diphtheria by Clysters.*—W. H. Vail, M.D., (*N. Y. Med. Record*, May 13, 1876) says: One case is reported—"a true malignant case." Patient was twenty two months old. Refusing to swallow, the rectum was used. Every four hours during the first week the remedies and food, with brandy, were injected together. When they were dejected, the clyster was immediately repeated, with ten drops of laudanum added, and in every instance the second injection was retained. Alvine dejections were normal and well formed. As the severe symptoms abated, the lavements were used every six hours. The rectum was thus used for three weeks. Recovery took place.

*Choleate of Soda as a Preventive of Gall Stones.* Wm. C. Dabney, M. D. (*Amer. Jour. Med. Sci.*, April, 1876.)—The article and its use, indicated above, were recommended by Prof. Schiff, of Florence, in 1874. Dr. Dabney has used it in three well-marked cases of biliary calculi diatheses, with extremely satisfactorily results. The attention of physicians who have under their care any patients suffering regularly from gall-stone colic, is called to this agent. It is used in 5 to 7-grain doses twice daily, and is a perfect solvent of the cholesterine and mucus which compose the "stones." It will prevent future attacks of colic, but will not relieve a fit of it when it is once precipitated.

*Bronchocele—Treatment by Injections of Alcohol.*—A male patient, æt. 28, native of Delaware county, New York, first noticed that his neck was enlarged twelve years ago, and the enlargement had been generally increasing since. The goitre was enormous, and most marked upon the right side. The case was to be treated by hypodermic injections of about twenty drops of alcohol, repeated according to effect produced. The injections were used with the idea of producing shrinking, as the result of the formation of new connective tissue in the enlarged organ.—*Bell. Hos. Med. Rec., N. Y.*

*Photography as a Means of Diagnosis.*—The photographic process is so acutely sensitive that it will often present vividly certain blemishes which the eye is incapable of detecting. Some years ago a lady had a photogroph taken of herself in which the face appeared covered with blotches which could not be seen in the original. The next day they became evident to the naked eye, and she finally died of small-pox. The photograph had proceeded farther than vision, and recognized, in advance, exceedingly delicate light-yellow stains.—*Vogel.—Photography and Chemistry of Light.*

*Sulphide of Calcium in Diabetes.*—In the *Medical Times and Gazette*, Dr. Scatliff describes a case of diabetes, of which he says: "He was, when I saw him, passing quarts of water night and day (sp. gr. 1028). At my request he tried Dr. S. Ringer's treatment (calc. sulphide one-eighth grain ter die), first in the form of powders mixed with sacch. lactis gr. iij, and afterward in pills. He immediately experienced great relief. Two days after he was passing only a normal quantity of water (sp. gr. 1023 5), and felt "quite comfortable." He had kept to his ordinary diet, and had not, in any way, avoided any amylaceous food at the time.—*Medical and Surgical Reporter.*

*Powder to Prevent Pitting After Variola.*—Dr. Pennavaria, of Ragusa, has succeeded in preventing pitting in several cases of variola by the use of a powder composed of four parts of flour of sulphur and one part of red precipitate. He was led to use it by the success he had had with it in eczema and acne. A thin layer of glycerine is first spread over the pustules as soon as they have reached the period of suppuration, and the powder is dusted over it. The glycerine secures the adhesion of the powder, which causes desiccation and the formation of a crust, and when this falls the skin is exempt from cicatrices.—*N. Y. Med. Record.*

*Cyanides in Acute Articular Rheumatism.*—In an article in the *Bulletin General de Therapeutique*, Dr. Luton, of Rheims, advocates the employment of cyanides in the treatment of acute rheumatism. He gives the history of ten cases, in all of which this treatment was strikingly successful; in one case the temperature was distinctly reduced by the cyanide. The salts he chiefly used were the cyanide of zinc, and the cyanide of potassium. The first is odorless and tasteless, and may be given suspended in mucilage or in pills, in doses of one and a half grains daily.—*Med. & Surg. Rep.*

*Acute Articular Rheumatism Treated with Salicylic Acid.*—A number of cases had been treated by administering six grains of the acid every two hours, and continuing it from six to ten grain doses three times a day, as the acute symptoms subsided. In every instance the symptoms had been markedly relieved, and, as a rule, quite promptly. This plan was adopted independent of the use of alkalies.—*Bellevn Hospital Medical Record, New York.*

*Sulphurous Acid as a Dressing for Recent Wounds.*—The following formula was used with great satisfaction in various parts of France during the late war :

R. Acidi sulphurosi..............................f.oz. ss,
Aquæ..........................................O i. M.

Apply in the form of lotion, or by means of an irrigator. This solution is particularly useful in wounds where there is much suppuration, or where cicatrization takes place slowly.—*New Remedies.*

*Incontinence of Urine.*—Mr. Brenchley writes to the *Practitioner* that he has seldom seen much good done in the above disease by belladona, iron, or bromide of potassium, but has met with much success with the following combination of ergot and iron:

R. Tinct. ergota.............................min. x.
Tinct. ferri. perchloridi...............min. v.
Spt. chloroformi........................min. v.
Infus. quassiæ......ad oz. i. ter die sumend.

—*New York Medical Record.*

*Meniere's Disease Treated by Quinine.*—Charcot has recently given large doses of quinine—from seven to fifteen grains a day for six or seven weeks—in two cases of Meniere's disease, with the result of enabling the patients to go about with security by themselves. He was led to give it in the hope that the ringing in the ears produced by the excessive doses of quinine might deaden the subjective noises in the ears dependent upon the disease.—*N. Y. Medical Record.*

*Chloroform in Hydrocele*—In the *Union Med de Canada,* Dr. Lubin, observing the frequent lumbar pain consequent upon the use of the ordinary injections in this affection, was led to use a formula similar to the following :

R. Tinct. iodinii comp......................fl.oz. iij
Chloroformi...............................fl.dr. ijss.

In a number of cases in which this mixture has been used, no pain whatever followed this injection.—*Med. & Surg. Rep.*

*Poulticing in Hæmoptyses.*—In an attack of hæmoptyses, a writer recommends poultices between the shoulders, at a temperature of about 122°, renewed every four hours.

*Coto Bark in Diarrhœa and Rheumatism.*—This new bark, from Bolivia, is said, by Professor Giete, of Munich, to be a specific against diarrhœa in its most diverse forms. He administers it in doses of 0·5 gramme of the fine powder, four or six times a day. Of the tincture, he usually gives ten minims every two hours. In Bolivia, whence the plant was sent, it is regarded as a remedy against rheumatism and gout.—*Med. & Surg. Rep.*

*Treatment of Epistaxis by the Internal Administration of Ergot.*—Epistaxis is sometimes difficult to arrest, especially in persons enfeebled by age, anæmia, etc. Dr. G. St. George states (*British Medical Journal*, Jan. 1, 1876) that he has found the liquid extract of ergot very serviceable where liquor ferri chloridi, plugging, etc., have been tried without avail. He relates three cases illustrative of its efficacy.

*Cold In the Head.*—Following prescription recommended to be taken as a snuff. One-quarter to one-half may be taken in 24 hours, with assurance of certain relief from coryza:

R. Hydrochlorate of Morphia.................gr. ij
Acacia Powder...............................dr. ij
Trisnitrate of Bismuth..... ............... dr. vj

Mix well.

*Animal Vaccination.*—A correspondent of the *British Medical Journal* very innocently asks the question, "if vaccination direct from the cow is now practiced. If so, by whom, and with what result?" Very evidently the medical reading of this gentleman has been quite limited.—*N. Y. Med. Rec.*

*Epistaxis*—Bleeding from the nose is often difficult to check, especially in persons enfeebled by age. Nearly every case may be controlled by giving fifteen drops fl. ext. ergot every fifteen minutes till checked, then every four hours for a day or two.—*Eclec. Med. Jour.*

*Cod-Liver Oil Injections.*—These are recommended, in the *Medical Times* for the oxyuris vermicularis, or seat worm. It may be readily dislodged from its favorite habitat in the rectum by the injection of two or three ounces of ol. morrhuae, re-repeated once or twice.—*Med. & Surg. Rep.*

*The Sialagogue Effect of Jaborandi Prevented by Hypodermic Injection of Morphia and Atropia.*—When jaborandi is to be administered, it has been customary to precede it with a hypodermic injection, consisting of ⅛ of a grain of the sulphate of atropia. This has been done for the purpose of preventing salivation, and has proved successful.—*Bellevue Hospital Medical Record, N. Y.*

*Toothache.*—A paste, made in the palm of the hand, by dropping on to a pinch of soda bicarbonate as much laudanum or wine of opium as the soda will take **up**, put into a cavity of the carious tooth, will often relieve the pain wholly.

*Painful Menstruation.*—R. Cincho Quinine. gr, lxxx; macrotin, gr, xviij; extract stramonium, gr vj; sacchari albi, gr, xxiv; Mix. Divide into 54 powders. Dose, one powder 3 times a day, commencing ten days before the expected period, continuing during its first day.—*Eclec. Med. Jour.*

*Chloral in Ozœna.*—The following has been used, and highly recommended, as a topical application in ozæna:

R—Chloral hydrat...................dr. ss.

Aq. puræ.........................oz. viij.—M.

*Chloral as an Antiseptic.*—Having recently had occasion to remove some shreds of membrane, which had remained in the uterus for a week after a miscarriage at three months, and had occasioned severe constitutional disturbance, we resorted to all the means at our disposal to rid our hand of the horrible stench imparted to it through the operation. In spite, however, oi most thorough washings with carbolic soap and subsequent applications of cologne water, the disagreeable odor remained, but was afterwards removed by a solution of chloral hydrate dr. j. to oz. iv. of water. We have never employed anything with nearly such satisfactory result in such cases.—*The Peninsular Journal of Medicine.*

*Rancid Oil of Maize in Skin Diseases.*—The *Centralblatt fur Med. Wiss.*, April 29th, 1876, contains an article by Professor Lombroso, recommending the above oil as an application in chloasurata and eczemata.—*Med. & Surg. Reporter.*

*Powder for Producing Ozone.*—In order to produce artificial ozone, Mr. Lender makes use of equal parts of peroxide of manganese, permanganate of potassium, and oxalic acid. When this mixture is placed in contact with water, ozone is quickly generated. For a room of medium size, two spoonsful of this powder, placed on a dish and occasionally diluted with water, would be sufficient. The ozone develops itself; it disinfects the surrounding air without producing cough.—*Medical Press and Circular, Juue 14th,* 1876.

*Cold Water Bath in Summer Complaint of Children.*—Dr. H. C. Wood, editor *Medical Times,* (Phila.,) writes: Any one who has seen, as I have this summer, the child on whom drugs had ceased to act, and was seemingly doomed to die, relieved in twelve hours by enforced cold bathing every three or four hours, will grant to Dr. Comegys the credit of having introduced one of the most life-saving improvements in modern infantile therapeutics. The sudden sweet sleep, replacing, after the bath, the fretful nights and days of unrest, is a thing never to be forgotten when once seen, and the arrest of diarrhœa is certainly no less remarkable.—*Medical Times.*

*Castor Oil.*—This nauseous drug can be easily taken, when administered in the following way: The glass should be first rinsed with moderately hot water, and then two drachms of hot water, one once of castor oil and one drachm of peppermint water, put in the order mentioned. This is easily swallowed, and leaves hardly any taste of the oil in the mouth.—*The Medical Record.*

*Daturia as a Substitute for Atropia.*—It has been proposed to substitute daturia, the alkaloid of the Jamestown weed, for atropia, as a mydriatic. The two alkaloids were formerly considered as identical, but their difference is now acknowledged. Daturia is three times as active as atropia, and its dose is therefore much less. When applied to the eye, it does not cause the pain and confusion of vision which follow the use of atropia. Its effects are more constant and persistent. Such are the statements recently published.—*Pacific Med. and Sur. Journal.*

*The Action of Coca.*—Since the writing of Sir R. Christison on his experience in the use of coca leaves as a means of preventing fatigue, several have experimented upon themselves. Among the latest who have published the results are two Irishmen—Walter Bernard, and J. R. Leebody, of Londonderry. Starting one night at midnight, after a day of unusual fatigue, they walked twenty-four miles, part of the way over a stony road, and ascended a steep hill over thirteen hundred feet high. Both men, in the course of the walk, chewed each two doses of forty grains of the leaves; and were decided in their opinions that they suffered less from fatigue or the effects of the severe exercise than they would have done had the chewing of the coca not been resorted to.—*Brit. Med. Jour., June 17th.*

*A Nut for Contagionists.*—Golwood is a town of 200 inhabitants, situated on the Bombay and Baroda Railway, in Hindostan. According to despatches received by the London *Times*, cholera made its appearance there on the 4th of June, the first case occurring at noon on that day. Before daybreak next morning there were fifty-seven deaths, and in three days half the inhabitants had perished. We should like some believer in the propagation of cholera by contagion, to explain this and similar sudden outbreaks.—*Pacific Med. and Sur. Journal.*

*Salicylic Mixture in Diphtheria.*—In the *Med. Cent. Zeitung*, April 26, Dr. Tenholt reports very flattering success in diphtheria, with a mixture of salicylic acid and lime water, two to two hundred. The throat is touched or gargled frequently with this. In two or three days all his cases recovered.—*Med. & Surg. Reporter.*

*Hæmoptysis Treated by Ergot—Report of Fifty Cases.*—Dr. J. Williamson (*London Lancet*, January, 1876,) reports fifty consecutive cases of hæmoptysis treated by ergot. Out of these the drug rapidly checked bleeding in forty-four cases. In the other six it failed, as did also gallic acid.—*Detroit Rev. of Medicine.*

